# Neural Representation of Valenced and Generic Probability and Uncertainty

**DOI:** 10.1523/JNEUROSCI.0195-24.2024

**Published:** 2024-06-12

**Authors:** Jae-Chang Kim, Lydia Hellrung, Marcus Grueschow, Stephan Nebe, Zoltan Nagy, Philippe N. Tobler

**Affiliations:** ^1^Zurich Center for Neuroeconomics, Department of Economics, University of Zurich, 8006 Zurich, Switzerland; ^2^Neuroscience Center Zurich, University of Zurich, Swiss Federal Institute of Technology Zurich, 8057 Zurich, Switzerland

**Keywords:** attention, information, motivation, punishment, reward

## Abstract

Representing the probability and uncertainty of outcomes facilitates adaptive behavior by allowing organisms to prepare in advance and devote attention to relevant events. Probability and uncertainty are often studied only for valenced (appetitive or aversive) outcomes, raising the question of whether the identified neural machinery also processes the probability and uncertainty of motivationally neutral outcomes. Here, we aimed to dissociate valenced from valence-independent (i.e., generic) probability (*p*; maximum at *p* = 1) and uncertainty (maximum at *p* = 0.5) signals using human neuroimaging. In a Pavlovian task (*n* = 41; 19 females), different cues predicted appetitive, aversive, or neutral liquids with different probabilities (*p* = 0, *p* = 0.5, *p* = 1). Cue-elicited motor responses accelerated, and pupil sizes increased primarily for cues that predicted valenced liquids with higher probability. For neutral liquids, uncertainty rather than probability tended to accelerate cue-induced responding and decrease pupil size. At the neural level, generic uncertainty signals were limited to the occipital cortex, while generic probability also activated the anterior ventromedial prefrontal cortex. These generic probability and uncertainty signals contrasted with cue-induced responses that only encoded the probability and uncertainty of valenced liquids in medial prefrontal, insular, and occipital cortices. Our findings show a behavioral and neural dissociation of generic and valenced signals. Thus, some parts of the brain keep track of motivational charge while others do not, highlighting the need and usefulness of characterizing the exact nature of learned representations.

## Significance Statement

Encoding the probability and uncertainty of outcomes is important for adaptive behavior. Here, we ask to what extent the brain represents probability and uncertainty regardless of whether the predicted outcomes are valenced (i.e., motivationally relevant) or generic (i.e., valence-independent). We dissociate generic from valenced variables by using not only cues that predict appetitive or aversive outcomes but also cues that predict neutral outcomes. Our data reveal distinct behavioral effects and largely separate neural representations of valenced and generic variables. For example, valenced probability activated more proximal parts of the medial prefrontal and occipital cortices whereas generic probability activated more distal parts. Thus, the representation of probability and uncertainty is multiplexed, allowing for tailored information processing according to computational needs and outcome properties.

## Introduction

Information on how likely or how uncertain a future outcome is enables organisms to adaptively prepare behavior and deploy attention before the outcome occurs, thus taking advantage of the predictive relation between cues and outcomes. Given their relevance for survival, one may argue that valenced outcomes (rewards and punishments) should be processed with priority and facilitate the formation of associations with predictive stimuli compared with neutral outcomes (Lang and Davis, 2006). Compatible with this notion, cues associated with larger rewards or punishments accelerate behavior more strongly than cues associated with smaller rewards or punishments in deterministic situations (Kahnt and Tobler, 2013; Kahnt et al., 2014). For nondeterministic associations, several, not mutually exclusive, theoretical accounts characterize the role of probability and uncertainty of valenced outcomes for behavior and learning. According to one theory (Mackintosh, 1975), more reliable relations between cues and valenced outcomes will elicit more attention and learning, such that the effects on behavioral read-outs of attention are maximal for probability *p* = 1 and minimal for *p* = 0. According to another theory (Pearce and Hall, 1980), uncertain predictors of valenced outcomes elicit the most attention, such that effects on behavioral read-outs of attention are highest at *p* = 0.5 and lowest for certain predictors (i.e., *p* = 0 and *p* = 1). Finally, the inverse of uncertainty, precision, may be relevant for attention to action rather than attention-based learning (Gottlieb, 2012). In these theories, the valenced nature of motivating outcomes drives attention. However, from the perspective of information processing, there is not an obvious reason to treat valenced outcomes differently from neutral outcomes, raising the question to what extent valenced probability and uncertainty differ from generic (nonvalenced) probability and uncertainty.

At the neural level, valenced probability and uncertainty signals have been identified not only in rodents and nonhuman primates (Esber et al., 2012; Roesch et al., 2012; Sharpe and Killcross, 2014, 2015; Pauli et al., 2015) but also in humans (Litt et al., 2011; Rothkirch et al., 2012; Kahnt et al., 2014; Metereau and Dreher, 2015; Uddin, 2015; Rigoli et al., 2016; Zhang et al., 2017a,b; Seeley, 2019). In particular, the medial prefrontal cortex (mPFC) has been associated with valenced probability (Sharpe and Killcross, 2014, 2015; Metereau and Dreher, 2015), whereas the amygdala has been associated with valenced uncertainty (Li et al., 2011; Esber et al., 2012; Roesch et al., 2012). However, because previous research used only valenced but not neutral outcomes, it remained unclear whether these brain areas encode valenced or generic probability and uncertainty. Moreover, as valenced probability and uncertainty are thought to underlie motivational salience (Kahnt and Tobler, 2017), regions associated with salience may also (differentially) process these variables. Assessing this possibility is important because the relations between salience and probability or uncertainty remain often underspecified in the neural literature. Of particular interest in this context is the insula (Menon, 2015; Uddin, 2015; Seeley, 2019), which together with the dorsal anterior cingulate cortex is thought to form the hub of the salience network. As one primary definition of salience is an absolute value (increasing with both aversiveness and appetitiveness of outcomes), it is not surprising that some regions, such as the nucleus accumbens (NAcc), have been associated with both salience and value processing (Bartra et al., 2013; Seeley, 2019). For value, previous reports (Sescousse et al., 2013) have demonstrated higher-level (more generic) value activating more anterior regions of the ventromedial prefrontal cortex than lower-level (less generic) value. Based on this, we hypothesized that a similar anterior (generic)–posterior (valenced) separation would hold for probability and uncertainty in the mPFC.

**Figure 1.**
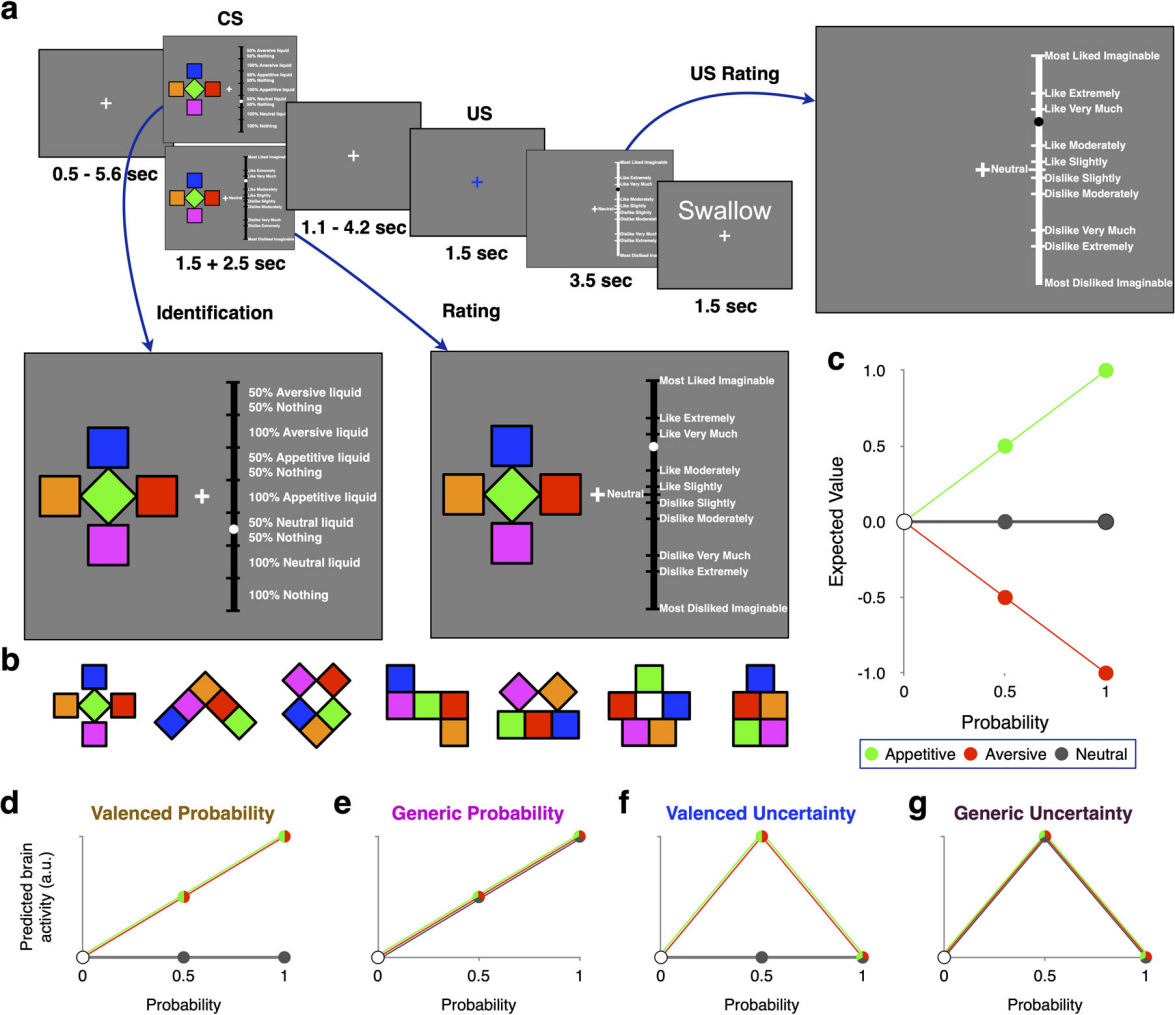
Trial structure and cues of Pavlovian conditioning task, expected cue value, and schematic of different forms of probability and uncertainty (signals). ***a***, Example trial of the main task. In each trial, one of seven previously learned cues (***b***) was presented. Each cue was probabilistically (*p *= 0, 0.5, or 1) associated with one of three different liquids (appetitive, aversive, neutral). First, participants were asked to either indicate the outcome associated with the cue or rate the pleasantness of the presented cue (bottom for all available options). The cue disappeared after 1.5  s, but participants could take another 2.5  s to respond. After a variable delay (1.1–4.2  s), the fixation cross turned blue, indicating the time of the outcome. Next, participants rated the pleasantness of the outcome. Finally, they rinsed with the neutral liquid and were instructed to swallow. Participants performed four runs per session (day) and two sessions, with each session comprising 56 trials. ***b***, Cues used as conditioned stimuli. The assignment of cues to conditions stayed constant within participants but was counterbalanced across participants. ***c***, Expected cue value. As probability increased in our experimental design, the expected value of cues increased for cues predicting appetitive liquids (green), decreased for cues predicting aversive liquids (red), and stayed unchanged for cues predicting neutral liquids (gray). The inclusion of both appetitive and aversive conditions allowed us to distinguish value from valenced probability, whereas the inclusion of the neutral condition allowed us to distinguish valenced from generic probability and uncertainty. ***d***, Valenced probability was standardized and changed similarly in the appetitive and aversive domains (see ***c*** for dissociation from expected value). This corresponds to motivational salience proposed by Mackintosh (1975). In this and all other figures of this kind, pie charts indicate that a condition contributes to multiple domains. ***e***, Generic probability varied also for cues predicting neutral liquid. ***f***, Standardized valenced uncertainty was maximal at an outcome probability of 0.5 for cues predicting valenced outcomes. This corresponds to the form of motivational salience proposed by Pearce and Hall (1980). With both valenced probability and valenced uncertainty, cues predicting neutral outcomes show no variation with probability. Note that the inclusion of *p *= 0 is necessary to dissociate uncertainty from (inverted) probability. ***g***, In contrast to valenced uncertainty, generic uncertainty was maximal at an outcome probability of 0.5 also for cues predicting neutral liquid.

Our novel Pavlovian conditioning task (Fig. 1*a*) used not only appetitive and aversive liquids but also neutral liquids. This allowed us to dissociate generic from valence-dependent probability and uncertainty, while controlling for mechanosensory stimulation. If probability or uncertainty as such were the only factors influencing brain activity and behavior, then we would expect similar effects of either variable for both neutral and valenced liquids. In contrast, if the valenced versus nonvalenced nature of predicted outcomes is relevant, then brain activity and behavior should process probability or uncertainty differentially when they concern valenced rather than neutral liquids.

## Material and Methods

### Participants

After a screening session [see below, Screening session and cue learning (Day 1)], 50 healthy, right-handed volunteers (25 females) participated in two functional magnetic resonance imaging (fMRI) sessions. Inclusion criteria were age between 18 and 40 years, near-perfect vision, and nonsmoking. Exclusion criteria were arm or hand injuries, metal implants in the body, large tattoos in the region of the head or neck, claustrophobia, current medication, and neurological, psychiatric, or eating disorders as ascertained by telephone-based screening. Nine participants were not analyzed further, because they did not correctly identify the meaning of cues (<60% of trials; *n* = 3), showed excessive head motion (*n* = 2) or experienced technical problems (*n* = 4). Accordingly, we present data from 41 participants (22.4 ± 0.43 years, mean ± SEM; 19 females). The study was approved by the Research Ethics Committee of the Canton of Zurich, and written informed consent was obtained from all participants before the experiment.

### Liquid delivery

For each participant, a custom-made juice machine delivered five liquids (two appetitive and two aversive liquids, all individually selected in the screening session, and one neutral liquid) through polytetrafluoroethylene tubing (inner diameter, 0.81 mm; outer diameter, 1.63 mm) connected to separate reservoirs and a one-way pump (LabDos, HiTec Zang, https://www.hitec-zang.de/). Liquid delivery was controlled with Psychtoolbox-3 (based on Matlab R2014b, MathWorks). The tubes were held together by a mouthpiece made of di-(2-ethylhexyl) phthalate (inner diameter, 4.8 mm; outer diameter, 8.0 mm), which the participants kept lightly between their lips. Each tube protruded the mouthpiece by ∼1 mm to prevent mixing of liquids inside the mouthpiece.

### Evaluation of liquids by bidding and rating

To measure the subjective value of each liquid, participants first received one drop of the liquid and then rated that liquid on a general labeled magnitude scale (gLMS) from “strongest imaginable dislike” to “strongest imaginable like” (Bartoshuk et al., 2004; Lim et al., 2009; Barrett and Simmons, 2015). In a second subjective value measure, participants provided a bid (b) on how much physical effort (quantified in percent of participant-specific maximal hand grip force, with 100% corresponding to the participant-specific maximum) they were willing to exert in order to obtain or avoid another drop of that liquid. Following a Becker–DeGroot–Marschak auction, the actual price of obtaining or avoiding another drop was determined by a uniformly distributed random draw *n* from (0, 100%). For appetitive (aversive) liquid, if *b* ≥ *n*, the participant received (avoided) another drop of liquid and exerted effort equal to *n* for 3 s. In contrast, if *b* < *n*, the participant received no additional drop of the appetitive liquid (had to endure another drop of the aversive liquid) but did not have to exert any effort (Becker et al., 1964). Ratings and bids were correlated (*r* = 0.66, *p* < 0.001). In case of discrepancy, we used bids to match the absolute value of appetitive and aversive liquids. During the screening session (Day 1), we screened 72 participants. In 22 out of these participants, we could not find two pairs of liquids with matched absolute values. These participants did not take part in the fMRI sessions.

### Study design

After the screening session outside the scanner, participants performed two main task sessions inside the scanner that were separated by 1–30 d (9.46 ± 1.32, mean ± SEM). Participants were asked not to eat or drink for at least 3 h before each session.

#### Screening session and cue learning (Day 1)

It is necessary to use both appetitive and aversive liquids to dissociate value (decreasing with probability of aversive outcomes, increasing with probability of appetitive outcomes) from valenced probability (increasing with probability of both aversive and appetitive outcomes). In the screening session, we selected and individualized four liquids to each participant's taste, such that the absolute subjective values of the appetitive and aversive liquids were similar. We used fruit juices (apple, *n* = 18; mango, *n* = 15; orange, *n* = 12; pineapple, *n* = 9; grape, *n* = 6) and milk drinks (vanilla, *n* = 4; strawberry, *n* = 10; chocolate, *n* = 8) as appetitive liquids (reward) and salty water (NaCl, 342 mM, *n* = 5; 684 mM, *n* = 12; 1.03 M, *n* = 10; 1.37 M, *n* = 6; 1.71 M, *n* = 8), bitter solutions [6-n-propylthiouracil (PROP), 0.032 mM, *n* = 3; 0.32 mM, *n* = 16; and 3.2 mM, *n* = 17], and sour juices (lemon, *n* = 3; lime, *n* = 2) as aversive liquids (punishment). The inclusion of neutral liquid conditions allowed us to identify generic probability and uncertainty signals and separate them from valenced probability and uncertainty signals. Distilled water with the main ionic components of saliva (KCl 25 mM, NHCO_3_ 2.5 mM) served as a neutral liquid. In a pilot study, participants indicated primarily that the solution was tasteless, with occasional reports of a slightly salty or metallic taste. In the main study, participants evaluated all liquids by bidding and rating.

Once the liquids were determined, participants learned to associate seven distinct visual cues with valenced, neutral, or no-liquid outcomes (drops of 0.2 ml occurring with probability *p* = 0, 0.5, or 1). The *p* = 0 cues are never followed by any liquid and by definition could not be associated with a particular valence domain (denoted with a white dot in the figures). We chose these probabilities to maximize the difference between probability and uncertainty and limit the number of cues participants had to learn). To control for basic visual properties of the cues, we used differently oriented and positioned combinations of the same five colored squares (Fig. 1*b*). Upon presentation of a cue, participants were asked to predict the outcome in this trial. The learning part consisted of two blocks. In the first block, each cue was presented 10 times in a row, while in the second block, each cue was presented 10 times in randomized order. Participants had to achieve an identification performance of at least 80% in the second block (chance level, 14.2%). If they failed to do so, the second block was repeated. Thereby, all participants reached an 80% accuracy (confirmed for both fMRI sessions). The cue–outcome associations were participant-specific, counterbalanced across participants, and remained the same in the main task. Thus, participants entered the scanner only after they successfully learned the cue–outcome associations.

#### Main task sessions (Days 2–3)

In the main task (Fig. 1*a*), each trial started with the presentation of one of the seven visual cues (Fig. 1*b*). Together with the cue, participants were asked to either indicate the outcome associated with the cue or rate the pleasantness of the presented cue on a gLMS (Fig. 1*a*). The cues always disappeared after 1.5 s, but participants had another 2.5 s to respond. After a variable delay (1.1–4.2 s), the fixation cross turned blue while the participants received either a 0.2 ml drop of liquid or no liquid according to cue probability. Next, participants rated the pleasantness of the outcome using a trackball device. Finally, they were instructed to swallow and rinse with the neutral liquid. In addition to fMRI data, we measured eye gaze, heart rate, and respiration during the entire scanning session and response times and pupil dilation in response to cues and outcomes. On each main task day, participants performed four blocks of 56 trials viewing each cue eight times. To limit habituation or sensitization, we used one pair of appetitive and aversive liquids in two of these blocks and the other pair in the remaining two blocks. The presentation order of the two pairs of liquids was counterbalanced across days.

### Neuroimaging

#### Data acquisition

Participants were scanned in a 3 T Achieva MRI scanner (Philips Healthcare) with a 32-channel receive-only head coil. Because the focus of our study was probability and uncertainty processing in ventral brain regions, particularly the ventral prefrontal cortex, striatum, insula, amygdala, and visual cortex, each run acquired 390 T2*-weighted echoplanar image (EPI; Schmitt et al., 2012) volumes with slice coverage limited to these regions while participants performed the task. For each volume, we acquired 33 slices in ascending order and the following parameters: voxel size, 2 × 2 × 2 mm^3^; field of view (FOV), 192 × 192 × 72 mm^3^; slice gap, 0.2 mm; repetition time (TR), 2,100 ms; echo time (TE), 27 ms; flip angle, 90°; matrix, 96 × 94. Before acquiring functional data, we collected a 2D multislice dual-echo gradient echo image (Haacke et al., 1999; TE: 4.6 and 6.9 ms). From these data, we computed a B_0_ field map, which during postprocessing we used to correct for distortions in the EPI data due to inhomogeneity in the magnetic field. To facilitate coregistration of the main fMRI data (Limbrick-Oldfield et al., 2019), we acquired a single additional whole-brain EPI volume (60 slices; voxel size, 2 × 2 × 2 mm^3^; FOV, 192 × 192 × 132 mm^3^; slice gap, 0.2 mm; TE, 27 ms; flip angle, 90°; matrix, 96 × 94) on both days. Finally, we acquired a T1-weighted high-resolution 3D turbo-field-echo anatomical image with a 1 mm isotropic resolution, an FOV of 256 × 256 × 170 mm^3^, a TR of 8 ms, and a TE of 3.7 ms.

#### Image preprocessing

We used Statistical Parametric Mapping (SPM, https://www.fil.ion.ucl.ac.uk/spm/software/spm12/) to preprocess the fMRI data with the following steps: slice time correction, motion correction, and magnetic field inhomogeneity correction. Next, coregistration of the fMRI data was accomplished in three steps: (1) all preprocessed fMRI data were aligned to the whole-brain EPI volume from each day; (2) the whole-brain EPI volume was aligned to the high-resolution anatomical image; and (3) the alignment parameters from Step 2 were applied to the actual fMRI data. Finally, we segmented the anatomical image into gray and white matter, DARTEL-normalized both the anatomical and the coregistered EPI images to MNI space, and spatially smoothed the EPI images using a three-dimensional Gaussian ﬁlter (6 mm full-width at half-maximum).

To model physiological noise, we used the PhysIO toolbox (Kasper et al., 2017) with the following parameters: cardiac pulsation (third-order Fourier expansions), respiration (fourth-order Fourier expansions), and cardiorespiratory interactions (first-order Fourier expansions). To remove spikes, we used the framewise displacement censoring method (Power et al., 2012) with a threshold of 0.6. If the number of censored volumes was bigger than 100 within a block (25.6% of the total number of volumes), the two blocks with the same pair of liquids were removed from further analysis. Moreover, participants were removed from further analysis if this procedure left less than four useable blocks (*n* = 2).

### Valenced and generic probability and uncertainty

In contrast to the expected cue value (Fig. 1*c*), valenced probability behaved similarly in the appetitive and aversive domains (Fig. 1*d*). Specifically, valenced probability increased with probability for appetitive and aversive liquids [(*p* = 1) > (*p* = 0.5) > (*p* = 0)], but not for neutral liquid (Fig. 1*d*). In contrast, generic probability increased also with probability of neutral liquids and did so similarly as with probability of appetitive and aversive liquids (Fig. 1*e*). Analogously, valenced uncertainty increased only for cues nondeterministically associated with valenced liquids [such that (*p* = 0.5) > (*p* = 0) = (*p* = 1)], approximating an inverted-U relation with probability. While uncertainty remained low for cues associated with probabilistically delivered neutral liquid in valenced uncertainty (Fig. 1*f*), it was high in generic uncertainty (Fig. 1*g*). Note that not only *p* = 0.5 but also *p* = 1 is relevant to dissociate probability from uncertainty coding. Moreover, it is conceivable that brain and behavior concurrently implement both probability and uncertainty (Pearce et al., 2010; Esber and Haselgrove, 2011).

### Behavioral and physiological data analysis

#### Subjective value

To assess whether the subjective value, measured by rating during the task, was explained by the expected (i.e., objective) value, we performed the following linear mixed effect regression (R version 3.6.2, lme4 package):

$$\begin{array}{rl} \mathrm{Rating}\;(\mathrm{subjective}\;\mathrm{value}) & =(\beta_{0\;}+b_{0j})+(\beta_{1\;}+b_{1j})\\ & \;\times \mathrm{Expected}\;\mathrm{value}+e_{j}, \end{array}$$

where *β*_0_ refers to the intercept, *β*_1_ refers to the model-specific fixed effect regression coefficient, *b*_0j_, and *b*_1j_ capture subject-specific random intercepts and slopes, and *e*_j_ refers to the residual error.

#### Response time and pupil dilation

Response times for the pleasantness ratings of the presented cue were converted to *z*-scores within subject and within block. Cue-related pupil dilations were standardized within subject and within block. They were defined as the difference in pupil size between the time of the response and pre-cue baseline (Murphy et al., 2014). Pupil size at the response was defined as the average pupil size from 250 ms before to 250 ms after the response, while baseline pupil size corresponded to the average pupil size during the 500 ms before cue onset (de Gee et al., 2017). We time-locked pupil data to the response because this is less susceptible to artifacts than time-locking to the peak dilation.

To assess whether the valenced probability and uncertainty explained response times and pupil dilation, we performed the following linear mixed effect regressions [the *α* threshold was set to 5% (two-tailed) for all analyses]:

$$\begin{array}{rl} & \mathrm{Standardized}\;\mathrm{dependent}\;\mathrm{variable}\\ & \;=(\beta_{0\;}+b_{0j})+(\beta_{1\;}+b_{1j})\times \mathrm{Subjective}\;\mathrm{value}+(\beta_{2\;}+b_{2j})\\ & \;\times \mathrm{Valenced}\;\mathrm{probability}+(\beta_{3\;}+b_{3j})\\ & \;\times \mathrm{Valenced}\;\mathrm{uncertainty}+e_{j}, \end{array}$$

where dependent variables refer to response time or pupil diameter, *β*_0_ captures the intercept, *β*_1_–*β*_3_ are model-specific fixed effect regression coefficients, *b*_0j_–*b*_3j_ capture subject-specific random intercepts and slopes, and *e*_j_ is the residual error.

We interrogated generic probability and uncertainty with a similar linear mixed effect regression, replacing valenced with generic probability and uncertainty:

$$\begin{array}{rl} & \mathrm{Standardized}\;\mathrm{dependent}\;\mathrm{variable}\\ & \;=(\beta_{0\;}+b_{0j})+(\beta_{1\;}+b_{1j})\times \mathrm{Subjective}\;\mathrm{value}\;+\;(\beta_{2\;}+\;b_{2j})\\ & \;\times \mathrm{Generic}\;\mathrm{probability}+(\beta_{3\;}+b_{3j})\\ & \;\times \mathrm{Generic}\;\mathrm{uncertainty}+e_{j}, \end{array}$$

To compare how well each variable explained behavior, we ran separate models:

$$\begin{array}{rl} & \mathrm{Standardized}\;\mathrm{dependent}\;\mathrm{variable}\\ & \;=(\beta_{0\;}+b_{0j})+(\beta_{1\;}+b_{1j})\times \mathrm{Subjective}\;\mathrm{value}+(\beta_{2\;}+b_{2j})\\ & \;\times \mathrm{Valenced}\;\mathrm{probability}+e_{j}, \end{array}$$

$$\begin{array}{rl} & \mathrm{Standardized}\;\mathrm{dependent}\;\mathrm{variable}\\ & \;=(\beta_{0\;}+b_{0j})+(\beta_{1\;}+b_{1j})\times \mathrm{Subjective}\;\mathrm{value}+(\beta_{2\;}+b_{2j})\\ & \;\times \mathrm{Generic}\;\mathrm{probability}+e_{j}, \end{array}$$

$$\begin{array}{rl} & \mathrm{Standardized}\;\mathrm{dependent}\;\mathrm{variable}\\ & \;=(\beta_{0\;}+b_{0j})+(\beta_{1\;}+b_{1j})\times \mathrm{Subjective}\;\mathrm{value}+(\beta_{2\;}+b_{2j})\\ & \;\times \mathrm{Valenced}\;\mathrm{uncertainty}+e_{j}, \end{array}$$

$$\begin{array}{rl} & \mathrm{Standardized}\;\mathrm{dependent}\;\mathrm{variable}\\ & \;=(\beta_{0\;}+b_{0j})+(\beta_{1\;}+b_{1j})\times \mathrm{Subjective}\;\mathrm{value}+(\beta_{2\;}+b_{2j})\\ & \;\times \mathrm{Generic}\;\mathrm{uncertainty}+e_{j}, \end{array}$$

We then computed the Akaike Information Criterion (AIC) for each case and compared it (ΔAIC) between valenced and generic variables.

To investigate the effects of probability and uncertainty in a domain-specific manner, we linearly regressed response times and pupil dilation separately on probability or uncertainty of appetitive, aversive, and neutral outcomes. This allowed us to assess whether behavior reflected the independent variables preferentially in some domains. Again, the regressions included subjective value:

$$\begin{array}{rl} & \mathrm{Standardized}\;\mathrm{dependent}\;\mathrm{variable}\\ & \;=(\beta_{0\;}+b_{0j})+(\beta_{1\;}+b_{1j})\\ & \;\times \mathrm{probability}\;\mathrm{of}\;\mathrm{appetitive}\;\mathrm{outcomes}+(\beta_{2\;}+b_{2j})\\ & \;\times \mathrm{Probability}\;\mathrm{of}\;\mathrm{aversive}\;\mathrm{outcomes}+(\beta_{3\;}+b_{3j})\\ & \;\times \mathrm{Probability}\;\mathrm{of}\;\mathrm{neutral}\;\mathrm{outcome}\;+e_{j}, \end{array}$$

$$\begin{array}{rl} & \mathrm{Standardized}\;\mathrm{dependent}\;\mathrm{variable}\\ & \;=(\beta_{0}+b_{0j})+(\beta_{1}+b_{1j})\\ & \;\times \mathrm{Standardized}\;\mathrm{uncertainty}\;\mathrm{of}\;\mathrm{appetitive}\;\mathrm{outcomes}+(\beta_{2\;}+b_{2j})\\ & \;\times \mathrm{Standardized}\;\mathrm{uncertainty}\;\mathrm{of}\;\mathrm{aversive}\;\mathrm{outcomes}+(\beta_{3}+b_{3j})\\ & \;\times \mathrm{Standardized}\;\mathrm{uncertainty}\;\mathrm{of}\;\mathrm{neutral}\;\mathrm{outcomes}+e_{j}, \end{array}$$

### Neuroimaging data analysis

We used SPM12 to build general linear models (GLMs) at the participants’ (first) level and then interrogated the resulting contrast images at the group (second) level. To detect neural activity related to subjective value, valenced probability, and valenced uncertainty, as well as to generic probability, uncertainty, inverse probability, precision, and pupil dilation, we estimated two GLMs. While GLM1 combined appetitive, aversive, and neutral domains in a single onset regressor that was parametrically modulated by valenced and generic variables, GLM2 modeled domains separately. This allowed us to interrogate and qualify the findings of GLM1. Specifically, the generic signals identified by GLM1 should show similar relations to probability or uncertainty for all three domains whereas for valenced signals the relation should be preferential and similar for the appetitive and aversive domains.

#### Participant-level analysis (first level)

For each participant and each block of trials, we modeled the following five main time periods as regressors within each trial and GLM: (1) cue presentation, (2) behavioral response to cue, (3) outcome, (4) behavioral response to outcome, and (5) swallow. We modeled each phase as an event with a duration equal to zero. Furthermore, we added the 26 regressors from the PhysIO toolbox for motion and physiological noise correction and separate regressors for each censored volume, as well as regressors for the difference in (1) eye position and (2) pupil diameter between the next TR and the current TR. Finally, we included a regressor for each block as a covariate. All these regressors entered two GLMs. In GLM1, we used six parametric modulators of the cue presentation regressor: (1) trial-specific ratings corresponding to the subjective value, (2) valenced probability, (3) valenced uncertainty, (4) generic probability, (5) generic uncertainty, and (6) pupil dilation. To allow all regressors to compete for independent components of explained variance, the orthogonalization of parametric regressors was turned off (Mumford et al., 2015).

In GLM2, we used three valence domain-specific onset regressors (appetitive, aversive, and neutral). We parametrically modulated each of these three regressors with (1) probability, (2) uncertainty, and (3) pupil dilation during the cue phase. Valenced probability or uncertainty signals require stronger relations to appetitive and aversive than neutral probability or uncertainty, but no significant difference between appetitive and aversive. Conversely, generic signals require that neutral probability or uncertainty is coded similarly as appetitive and aversive probability or uncertainty. As the separation of probability from uncertainty requires the use of all three probability levels (*p* = 0, *p* = 0.5, *p* = 1), we randomly assigned the *p* = 0 trials to the three onset regressors (note that this was not an issue in GLM1, where every condition entered all parametric modulators).

#### Group analysis (second level)

To identify regions where activity related to subjective value, valenced probability, valenced uncertainty, generic probability, generic uncertainty, and pupil dilation, we used flexible factorial designs that included the contrast images obtained for all cue-related parametric modulators from GLM1. We interrogated each parametric modulator using contrast images that we obtained by putting a 1 on the respective parametric modulator on the first level. On the second level, we also included regressors for day and participant to account for between-day and between-participant variability. We report whole-volume results (*p* < 0.05, cluster-level family-wise error (FWE)-corrected; cluster-inducing voxel-level threshold *p* < 0.001). For subjective value processing, we also report activation in the NAcc, an a priori region of interest (*p* < 0.05, peak-level FWE corrected within the region of interest). We specified the NAcc with the CIT168 Subcortical Atlas (Pauli et al., 2018). Exploratory analyses revealed no significant relation between activity in this ROI and valenced or generic probability or uncertainty. To extract activity from regions surviving whole-brain correction, we ran leave-one-subject-out analyses, in which we determined the peak coordinates for an activated cluster in *n*-1 participants and then extracted activity from a sphere with a radius = 4 mm in the left-out participant.

## Results

We investigated both valenced and generic variables with regressions explaining behavioral and neural responses to stimuli. A potential issue with this approach is multicollinearity, which would make it hard to unequivocally interpret the estimates associated with related regressors. To quantify the severity of the problem, we calculated the variance inflation factors (VIFs) per run and asked whether they exceeded the conservative threshold of 5 (O’Brien, 2007). The observed VIFs of 1.82 for valenced probability, 2.68 for valenced uncertainty, 1.71 for generic probability, and 2.57 for generic uncertainty were well below 5. Thus, actual collinearity among the different models was no cause for concern.

### Behavioral results

To quantify the subjective value of cues, participants rated each cue (Fig. 1*a,b*; see Materials and Methods, Subjective value). We also measured cue-induced response times of ratings and cue-induced pupil dilations (see Materials and Methods, Response time and pupil dilation), which have been associated with stimulus-related attention and arousal (Mathôt, 2018; Unsworth et al., 2018). It is worth keeping in mind though that the value of predicted outcomes also affects cue-induced responding (Kahnt and Tobler, 2013). The concurrent use of appetitive and aversive outcomes allowed us to distinguish between motivational salience (Mackintosh, 1975), which commonly increases with probability for both domains and value, which increases with probability in the appetitive domain but decreases in the aversive domain (Fig. 1*c*).

#### Task validation: subjective values of cues

**Figure 2.**
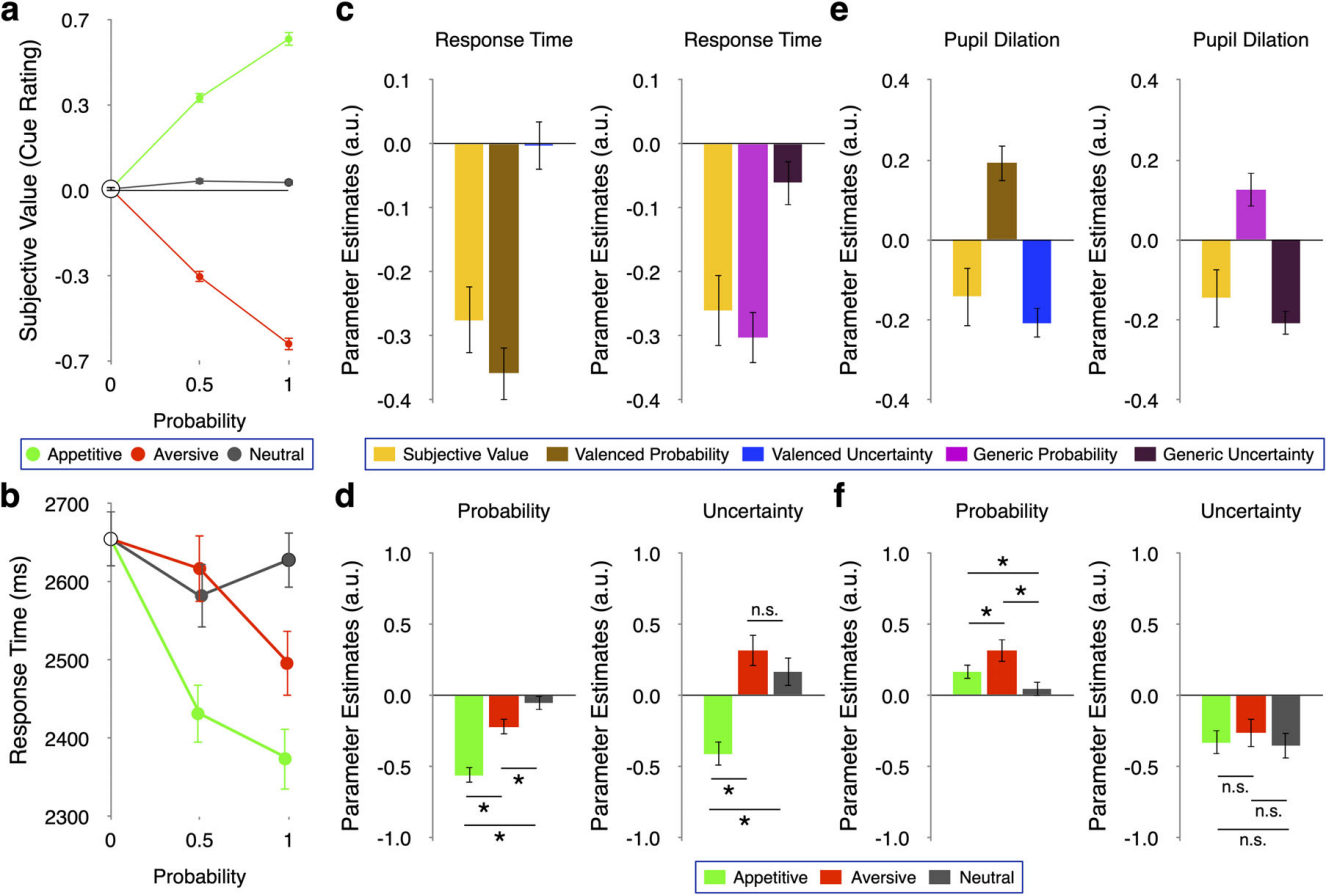
Cue rating, response times, and pupil dilation. ***a***, Subjective cue values. Cue ratings during the task were averaged over all participants. Note the close correspondence with expected value in Figure 1*c*. ***b***, Raw response times. As probability increased, response (i.e., the combination of reaction and movement) times decreased, particularly for stimuli associated with valenced liquids (appetitive, green; aversive, red) rather than neutral liquid (gray). This pattern was expected (Kahnt & Tobler, 2013) and reflects the combination of valenced probability (more appetitive and more aversive stimuli both have accelerating effects) and value (more appetitive stimuli reduce response times whereas more aversive stimuli increase them). Because participants indicated their responses on a slider, movement times were relatively long in our paradigm. The starting position of the cursor was pseudorandomized across trials, resulting in similar movement distances across conditions. Participants reached similar absolute ratings in the appetitive domain more quickly than in the aversive domain despite having to overcome geometrically slightly longer distances in the appetitive domain because of the asymmetry in the general labeled magnitude scale (Lim et al., 2009). Accordingly, faster responses in the appetitive than the aversive domain cannot be explained by movement time (but are compatible with the difference in subjective value between appetitive and aversive domains). ***c***, Response times decreased with valenced probability (left; *β*=−0.35, *t*_(43)_= −8.78, *p *< 0.001), subjective cue value (left; *β*=−0.27, *t*_(36)_= −5.22, *p *< 0.001), and generic probability (right; *β*=−0.30, *t*(75) = −7.65, *p *< 0.001). In contrast, neither valenced (left; *β *= −0.002, t_(39) _= −0.06, *p *= 0.95) nor generic (right; *β*=−0.06, *t*_(49)_= −1.78, *p *= 0.081) uncertainty showed a significant relation to response times. ***d***, Domain-specific analyses. Left, Both appetitive and aversive but not neutral probability accelerated responses. Right, While appetitive uncertainty accelerated responses, aversive uncertainty slowed them down, and neutral uncertainty had no significant effect. ***e***, Left, Valenced probability was positively associated with pupil size (*β*=0.19, *t*_(45) _= 4.29, *p *< 0.001), whereas valenced uncertainty (*β*=−0.14, *t*_(41) _= −3.76, *p *< 0.001) and, to a lesser degree, subjective value (*β*=−0.14, *t*_(41) _= −1.96, *p *= 0.057) showed a negative association. Right, A similar pattern emerged for generic variables, with generic probability enlarging (*β*=0.13, *t*_(185) _= 3.06, *p *< 0.01) and generic uncertainty decreasing pupil size (*β*=−0.12, *t*_(74)_=−4.24). ***f***, Domain-specific analysis. Left, Probability in all three domains increased pupil size, but the effects of appetitive and aversive probability were stronger than those of neutral probability. Right, For uncertainty, all three domains decreased pupil size, compatible with pupil diameter reflecting generic precision. *p < 0.05. Error bars represent ±1 standard error of the mean.

By design, the expected value of cues increased with probability of appetitive liquids, decreased with probability of aversive liquid, and remained unchanged as the probability of neutral liquids increased (Fig. 1*c*). The subjective cue evaluations followed a similar three-fold pattern (Fig. 2*a*). Accordingly, the association between subjective (ratings) and objective (expected) cue value was strong (*b* = 60.09, *t*_(41)_ = 24.82, *p* < 0.001). Importantly, the absolute ratings of cues predicting aversive liquids did not significantly differ from the ratings of cues predicting appetitive liquids (*t*_(40)_ = −1.16, *p* = 0.25 for probability = 0.5; *t*_(40)_ = 0.45, *p* = 0.65 for probability = 1). These results suggest that the absolute value of cues predicting appetitive liquids was matched to the absolute value of cues predicting aversive liquids.

#### Response times and pupil dilations modulated by probability and uncertainty

We first considered response times. Inspection of the raw data (Fig. 2*b*) suggested that cues associated with no or neutral liquid accelerate responses less than cues associated with aversive or appetitive liquid. Thus, in line with previous research (Peck and Salzman, 2014), the valenced outcomes appear to exert stronger motivating functions than neutral outcomes. To assess this appearance statistically, we regressed response times in all trials and domains first on valenced variables and then on generic variables (including subjective value in both analyses), using separate linear mixed effects models. Response times decreased as valenced and generic probability (as well as subjective value) increased (Fig. 2*c*). However, model comparison showed that valenced probability explained response times better than generic probability (response times, ΔAIC = 140).

Domain-specific analyses (Fig. 2*d*, left) confirmed a significant relation of response times for appetitive (*β* = −0.60, *t*_(51)_ = −10.11, *p* < 0.001) and aversive (*β* = −0.22, *t*_(55)_ = −4.01, *p* < 0.001), but not for neutral probability (*β* = −0.05, *t*_(53)_ = −0.99, *p* = 0.33). Direct comparison of *β* estimates indicated that the accelerating effect of probability was more pronounced for appetitive (paired *t* test, *t*_(40)_ = −10.46, *p* < 0.001) and aversive (paired *t* test, *t*_(40)_ = −3.94, *p* < 0.001) compared with neutral probability. These findings corroborate a preferential relation of response times with valenced rather than neutral probability.

In contrast to valenced probability, valenced uncertainty showed no significant effect on response times (Fig. 2*c*, left). Moreover, although generic uncertainty had a trend-level accelerating effect on response times (Fig. 2*c*, right), it failed to explain them better than valenced uncertainty (ΔAIC = −2). Domain-specific analyses revealed an accelerating effect of appetitive uncertainty (Fig. 2*d*, right; *β* = −0.41, *t*_(41)_ = −4.84, *p* < 0.001). In contrast, aversive uncertainty slowed responding (*β* = 0.32, *t*_(40)_ = 2.93, *p* < 0.01), and the effect of neutral uncertainty on response times was not significant (*β* = 0.17, *t*_(44)_ = 1.68, *p* = 0.10). These data suggest that valence matters also for uncertainty, such that appetitive uncertainty appeared to be motivating whereas aversive uncertainty appeared to be demotivating.

Regarding pupil dilation, increasing valenced probability resulted in larger pupil size (Fig. 2*e*, left), while larger valenced uncertainty and, to a lesser degree, larger subjective value were associated with smaller pupil size (Fig. 2*e*, left). A similar pattern emerged also for generic variables (Fig. 2*e*, right). However, domain-specific analyses (Fig. 2*f*) revealed that neutral uncertainty affected pupil diameter strongly (*β* = −0.35, *t*_(49)_ = −3.93, *p* < 0.001), whereas neutral probability did so only weakly (*β* = 0.10, *t*_(77)_ = 2.13, *p* < 0.05). Accordingly, valenced probability explained pupil diameter better than generic probability (ΔAIC = 30) whereas generic uncertainty had more explanatory power compared with valenced uncertainty (ΔAIC = 20). Note that uncertainty showed a negative association with pupil size in all three domains. In other words, pupil size correlated with generic precision in our task. Together, the pupil data provide some evidence that the certainty with which cues predict outcomes can affect some forms of behavior in a valence-independent manner.

### Neural results

We analyzed the neural data in a parametric and a nonparametric fashion and with increasing granularity. First (GLM1), we interrogated a model with one onset regressor and valence-dependent and valence-independent (as well as subjective value and pupil) parametric modulators. Thus, all cues were modulated by all parametric modulators and contributed to all parametric modulators. Next (GLM2), we asked whether the findings of GLM1 were driven by a particular domain, modeling appetitive, aversive and neutral onsets separately, and modulating each of these regressors with probability and uncertainty. To account for the fact that *p* = 0 trials are required for all three onset regressors in GLM2, in a separate analysis, we randomly assigned a third of the *p* = 0 trials to the three onset regressors in variants of GLM2 and averaged the results.

#### Validation: the ventral striatum and ventromedial prefrontal cortex process subjective value

**Figure 3.**
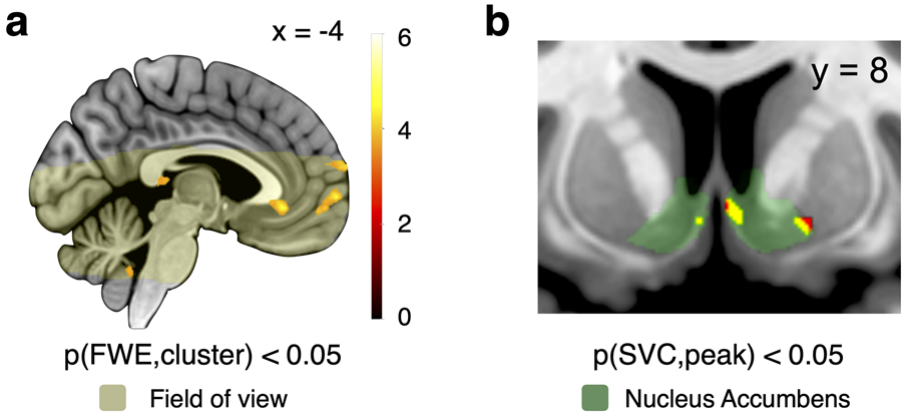
Field of view and activity related to subjective value. ***a***, The field of view covered the ventral brain (light yellow) with 2  mm isotropic resolution and included regions associated with processing salience and value such as the ventral striatum, insula, ventromedial prefrontal cortex (vmPFC), and amygdala. Activity in vmPFC regions correlated with subjective value at the time of cue presentation [e.g., posterior vmPFC cluster: *t *= 6.79; whole-brain family-wise error rate (FWE) cluster-level corrected, *p *< 0.05; cluster-inducing voxel threshold, *p *< 0.001]. ***b***, Regions within the nucleus accumbens (green) correlated with subjective value (e.g., right medial cluster: *t *= 4.55, *p *< 0.05, peak-level FWE small-volume corrected). The color bar represents *t* values for parametric subjective cue value contrast.

As a sanity check for the neural data produced by our task, we first identified brain regions showing increasing activity with the subjective value of the cues. We constructed trial-by-trial parametric modulators based on each participant's ratings and regressed blood oxygen level-dependent (BOLD) signals against these trial-wise estimations in GLM1 to compute the strength with which the activity at each voxel processed value (see Materials and Methods, Neuroimaging data analysis). We found value processing signals in the ventromedial prefrontal cortex (vmPFC; *t* = 6.79; whole-brain FWE cluster-level corrected, *p* < 0.05, cluster-forming threshold: *p* < 0.001; Fig. 3*a*; Table 1; at the whole-brain level, only the occipital cortex showed an overlap of subjective value and any probability or uncertainty signals), a canonical region associated with processing subjective value (Bartra et al., 2013). In addition to the whole-brain analysis, we also examined the effect of subjective value on BOLD activity in the NAcc, a second canonical value-processing region (Bartra et al., 2013). NAcc activity increased with subjective value (right NAcc; *t* = 4.55, *p* < 0.05, peak-level FWE small-volume corrected in the NAcc; Fig. 3*b*; Table 1). Thus, prime value-processing areas showed increasing activity as cue value increased. These findings provide face validity for our task.

**Table 1.** Brain regions encoding subjective value (GLM1)

Contrast	Region	Cluster size (# voxels)	Mean *t* statistic	Peak MNI coordinates		
				*x*	*y*	*z*
Subjective value
	Cuneus_R (occipital cortex)	386	8.77	22	−92	12
	Olfactory_R (vmPFC)	127	6.79	2	20	−4
	Frontal_Med_Orb_R (vmPFC)		4.30	2	42	−10
	Right cerebral white matter^a^	61	6.38	20	30	10
	Frontal_Sup_2_R (lPFC)	65	5.68	30	64	18
	Frontal_Sup_Medial_L (mPFC)	125	5.10	−14	62	12
	Frontal_Sup_Medial_R (mPFC)		4.21	8	72	12
	Rolandic_Oper_R	190	5.03	68	−10	12
	Right cerebral white matter^a^		3.86	50	−2	22
	Frontal_Sup_Medial_L	115	4.88	−2	64	2
	Cerebellum	71	4.81	2	−46	−40
	Left cerebral white matter^a^	136	4.27	0	−28	10
	Nucleus accumbens^b^		4.55	2	8	−6
			4.01	6	12	−8
			4.40	18	6	−10

Abbreviations: MNI, Montreal Neurological Institute; vmPFC, ventromedial prefrontal cortex; lPFC, lateral prefrontal cortex; mPFC, medial prefrontal cortex.

a Label from Harvard-Oxford Atlas (region not defined in the AAL Atlas 3).

b Small volume corrected within anatomical ROI defined by the CIT168 Subcortical Atlas (Pauli et al., 2018). Apart from the signal in the nucleus accumbens, all activations are whole-brain family-wise error rate (FWE) corrected at the cluster-level corrected, *p* < 0.05; cluster-inducing voxel threshold *p* < 0.001. Apart from b (see below), the gray matter labels are from the Automated Anatomical Labelling (AAL) Atlas 3 (Rolls et al., 2020).

#### Valenced probability signals in the mPFC, insula, and temporal and occipital cortices

**Figure 4.**
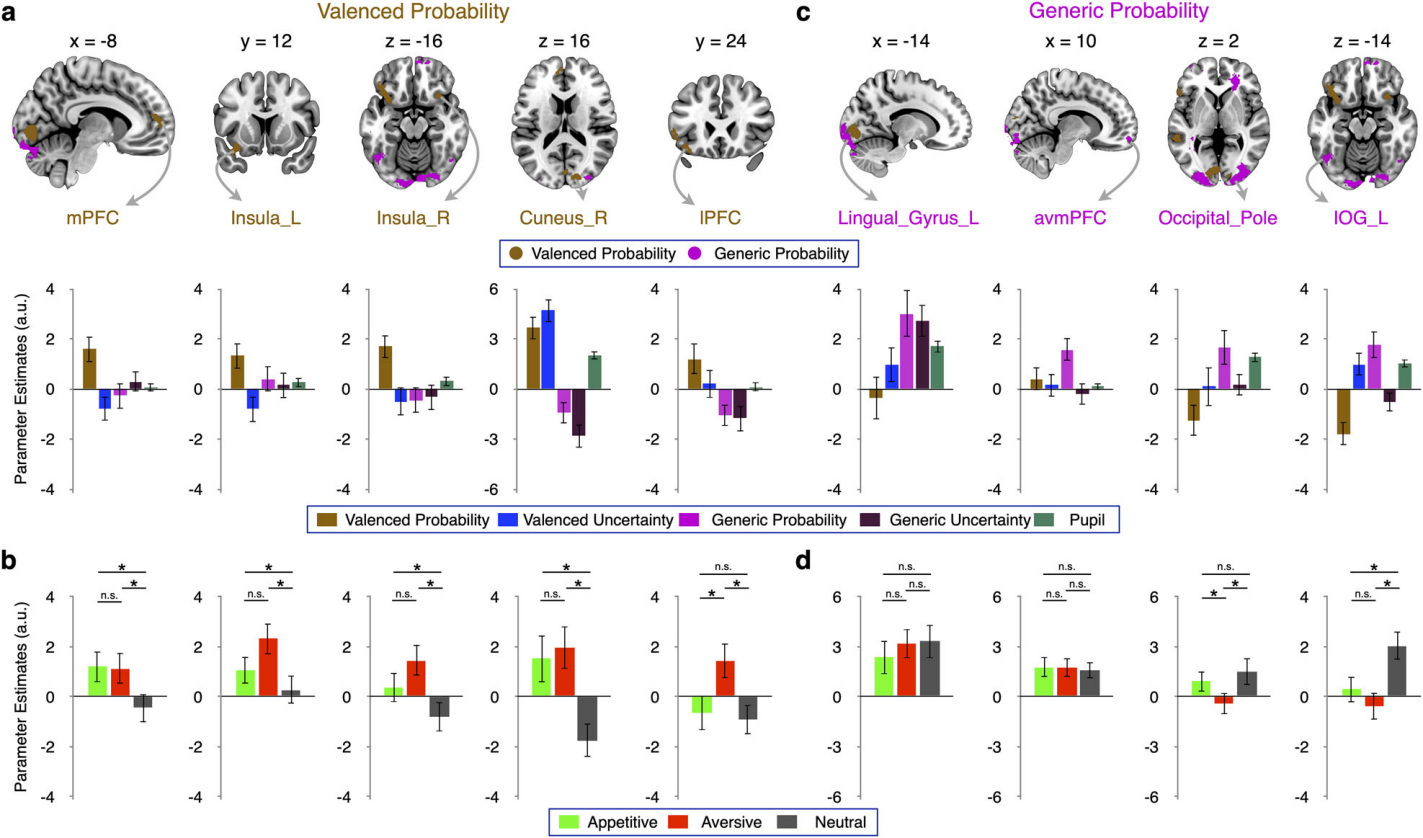
Neural representation of valenced and generic probability. ***a***, Valenced probability signals (identified by a parametric modulator formed according to Fig. 1*d* in GLM1). The medial prefrontal cortex (mPFC, *t *= 5.39), bilateral ventral anterior insula (left, *t *= 5.59; right, *t *= 5.87), cuneus (*t *= 10.66), and lPFC (*t *= 4.92) activity increased with valenced probability (brown regions and bar plots). ***b***, Separate analysis of appetitive, aversive, and neutral probability in GLM2 specified the findings of GLM1. Proper valenced probability signals require (similar) encoding of appetitive and aversive probability but not of neutral probability. These requirements were not imposed by GLM1 but met by mPFC, insula, and cuneus. In contrast, the lPFC region primarily encoded aversive probability. ***c***, Generic probability signals. GLM1 identified generic probability coding (using a parametric modulator formed according to Fig. 1*e*) in the lingual gyrus (*t *= 5.59), the anterior vmPFC (avmPFC; *t *= 5.49), occipital pole (*t *= 5.10), and inferior occipital gyrus (*t *= 4.78). All tests of parametric modulations against zero in ***a*** and ***c*** were whole-brain FWE cluster-corrected (*p *< 0.05; cluster-forming threshold, *p *< 0.001). Direct whole-brain comparisons (i.e., not on the extracted data) between parametric modulators showed a statistically (*p *< 0.001, uncorrected) stronger relation of activity to valenced than generic probability in the regions shown in ***a*** and to generic than valenced probability in the regions shown in ***c***. ***d***, Proper generic probability signals require similar relations of brain activity to appetitive, aversive, and neutral probability. GLM2 showed that this requirement (not imposed by GLM1) was met by the lingual gyrus and avmPFC but not by the occipital pole (which encoded primarily appetitive and neutral uncertainty) and the inferior occipital gyrus (which encoded primarily neutral uncertainty). *p < 0.05, n.s., not significant. Error bars represent the standard error of the mean.

To localize brain regions involved in processing valenced probability and uncertainty, we used trial-by-trial parametric modulators that varied with the probability or uncertainty with which cues predicted valenced but not neutral outcomes. We then regressed BOLD signals against these trial-wise parameters in GLM1 (see Materials and Methods, Neuroimaging data analysis). We found whole-brain FWE cluster-corrected (*p* < 0.05; cluster-inducing voxel-level threshold, *p* < 0.001) valenced probability signals (Fig. 4*a*) in the bilateral ventral anterior insula (left, *t* = 5.59; right, *t* = 5.87) and mPFC (mPFC; *t* = 5.39). Other regions showing valenced probability signals included the lingual gyrus (*t* = 10.66) and lPFC (*t* = 4.92; Table 2). Direct comparisons within SPM (i.e., not on extracted parameter estimates, avoiding double-dipping) revealed that the relation of neural activity with valenced probability was significantly stronger than with generic probability within all these regions (*p* < 0.001, uncorrected). Thus, in line with the traditional framework of motivational salience, parts of the brain appear to process particularly the probability of valenced events rather than probability as such.

**Table 2.** Brain regions showing valenced probability and generic probability signals (GLM1)

Contrast	Region	Cluster size (# voxels)	Mean *t* statistic	Peak MNI coordinates		
				*x*	*y*	*z*
Valenced probability	Lingual_L (occipital cortex)	544	10.66	−8	−84	−6
	Cuneus_L (occipital cortex)		4.63	4	−80	14
	Cuneus_R (occipital cortex)	185	8.40	14	−90	16
	Temporal_Mid_L	155	6.06	−54	−42	2
	Temporal_Inf_L		3.61	−58	−28	−18
	Insula_R (ventral anterior insula)	55	5.87	28	18	−16
	Insula_L (ventral anterior insula)	256	5.59	−32	12	−16
	OFCpost_L (lOFC)		4.49	−40	32	−16
	Frontal_Sup_Medial_L (mPFC)	125	5.39	−8	54	12
	Temporal_Mid_L	85	5.04	−54	2	−34
	Frontal_Inf_Tri_L (lPFC)	84	4.92	−52	24	4
Generic probability	Occipital_Mid_L (MOG)	1,293	10.19	−24	−96	4
	Lingual_L (occipital cortex)		5.59	−14	−94	−16
	Cerebellum_Crus2_L		4.60	−10	−84	−34
	Occipital_Mid_R (MOG)	1,308	7.45	34	−94	6
	Fusiform_R		5.25	28	−84	−14
	Occipital pole^a^		5.10	10	−104	2
	Right cerebral white matter^a^	363	7.33	24	38	0
	Left cerebral white matter^a^	647	5.66	−36	−40	−4
	Cerebellum_Crus2_L		5.01	−46	−70	−38
	Occipital_Inf_L		4.78	−44	−58	−14
	Frontal_Med_Orb_R (avmPFC)	101	5.49	8	62	−12
	Cerebellum_Crus1_R	217	4.66	48	−66	−26
	Frontal_Mid_2_L (lPFC)	63	4.56	−40	58	−2
	Cerebellum_Crus2_R	72	4.47	16	−90	−32

All activations are whole-brain family-wise error rate (FWE) corrected at the cluster-level corrected, *p* < 0.05; cluster-inducing voxel threshold *p* < 0.001. Apart from a (see below), the gray matter labels are from the Automated Anatomical Labelling (AAL) Atlas 3 (Rolls et al., 2020).

Abbreviations: MNI, Montreal Neurological Institute; lOFC, lateral orbitofrontal cortex; mPFC, medial prefrontal cortex; lPFC, lateral prefrontal cortex; MOG, middle occipital gyrus; avmPFC, anterior ventromedial prefrontal cortex.

c Label from Harvard-Oxford Atlas (region not defined in the AAL Atlas 3).

Next, we used GLM2 to ask whether the valenced probability signals identified by GLM1 arose similarly for the probability of appetitive and aversive outcomes and more strongly for the probability of either of these valenced outcomes than of neutral outcomes (Fig. 4*b*). In line with a proper valenced probability signal, the mPFC, right insula, and occipital activity showed no significant difference between appetitive and aversive probability but statistically stronger coding of either of these probabilities than of neutral probability. Thus, these regions were not driven by one domain alone and, importantly, encoded the probability of appetitive and aversive outcomes more strongly than the probability of neutral outcomes, in line with a formal valenced probability signal. Separate analyses of valenced (i.e., combining appetitive and aversive) probability and neutral probability corroborated this conclusion (data not shown). However, note that lPFC activity showed a preferential relation to aversive probability, with significant differences to both appetitive and neutral probability, reinforcing the importance of considering valence domains separately.

#### Ventral prefrontal and occipital regions process generic probability

To examine generic probability, we searched for probability-related signals that, unlike valenced probability, increased with probability also for cues predicting neutral liquid (Fig. 1*e*). Accordingly, we constructed a trial-by-trial parametric modulator that commonly increased with probability in all three domains and regressed BOLD activity against it in GLM1 (see Materials and Methods, Neuroimaging data analysis). We identified whole-brain FWE cluster-corrected (*p* < 0.05; cluster-inducing voxel-level threshold, *p* < 0.001) generic probability signals [Fig. 4*c*, the anterior vmPFC (*t* = 5.49) and bilateral occipital cortex (lingual gyrus, *t* = 5.59; occipital pole, *t* = 5.10; inferior occipital gyrus (IOG), *t* = 4.78); Table 2]. Direct comparisons showed that the positive relation of neural activity to generic probability was significantly stronger in all these regions than the relation with valenced probability (*p* < 0.001, uncorrected).

Interrogating the findings of GLM1 with GLM2 showed that the requirements of a proper generic probability signal were met by the lingual gyrus and avmPFC. Activity in these regions was related to not only appetitive and aversive probability but also neutral probability, without any significant difference between domains (Fig. 4*d*). Separate analyses of valenced and nonvalenced probability confirmed that avmPFC and occipital activity increased with probability of both types of outcomes, in line with a generic probability signal (data not shown). The frontal and occipital regions encoding generic probability were both more distal from the center of the brain in the anteroposterior direction than the medial prefrontal and occipital regions encoding valenced probability described above (Fig. 4*a,c*). As one would expect, activity encoding the probability of neutral outcomes was significantly stronger in the two regions processing generic probability than in the respective two nearby valenced probability regions (avmPFC vs mPFC, *t* = 3.40, *p* = 0.002; lingual gyrus vs cuneus, *t* = 3.65, *p* < 0.001). Together, these data suggest that ventral brain regions process generic probability over and above valenced probability.

Using GLM2 for closer inspection of the other occipital regions identified by GLM1 as encoding generic probability showed little relation to aversive probability (occipital pole) and preferential relation to nonvalenced probability (IOG; Fig. 4*d*). The peak of the inferior occipital region was located more laterally (*x* = −44) than the peak of the lingual gyrus region coding generic probability proper (*x* = −14). Thus, GLM2 revealed that the apparent generic probability signal in lateral occipital regions was driven specifically by the probability of neutral outcomes.

#### Valenced uncertainty signals in the insula, lOFC, and occipital cortex

**Figure 5.**
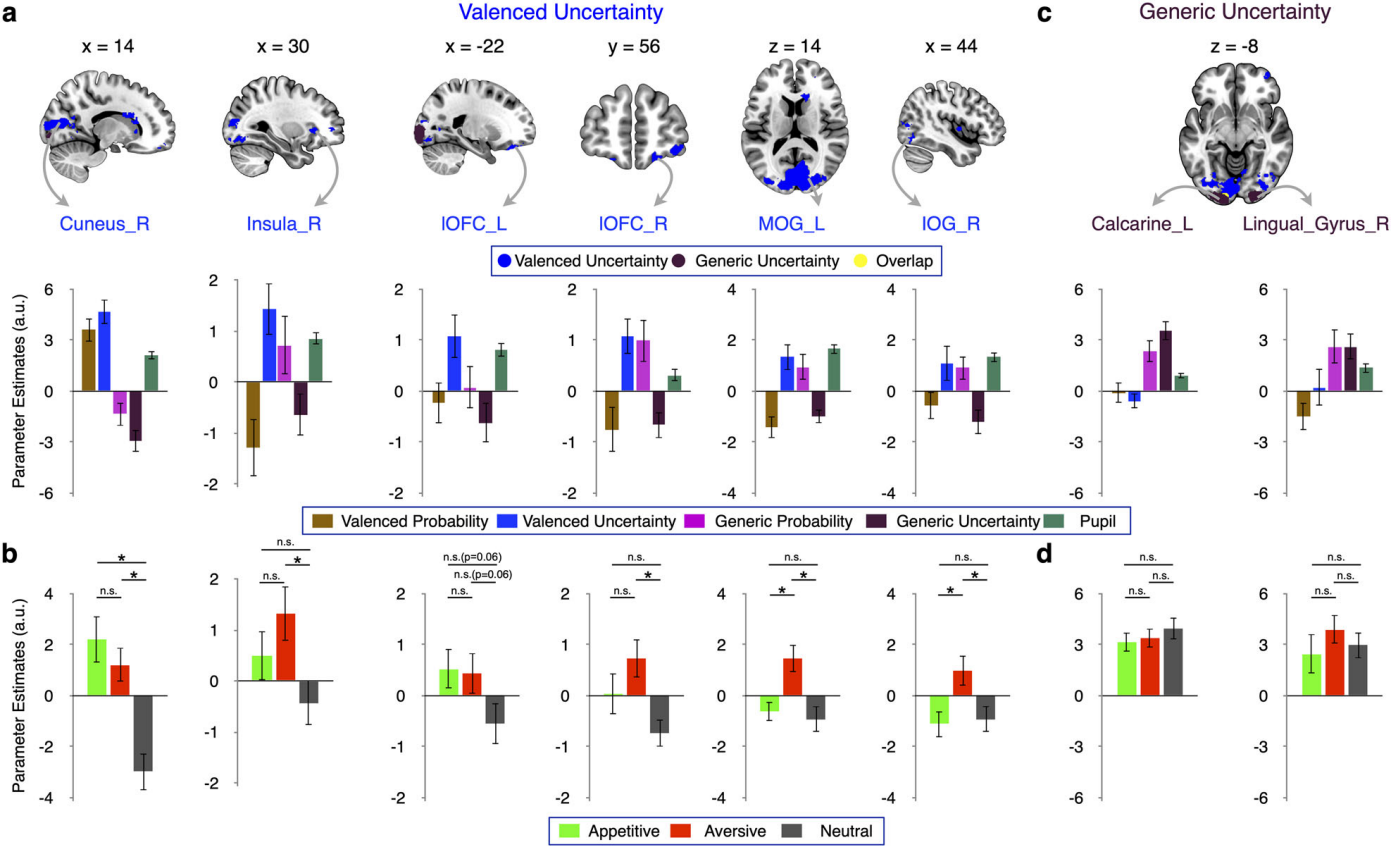
Neural representation of valenced and generic uncertainty. ***a***, Valenced uncertainty signals (identified by a parametric modulator formed according to Figure 1*f* in GLM1) in the cuneus (*t *= 13.03), right dorsal anterior insula (*t *= 6.01), bilateral regions in the lateral orbitofrontal cortex (left, *t *= 5.96; right, *t *= 5.06), middle occipital gyrus (MOG; *t *= 5.36), and IOG (*t *= 4.39). ***b***, Separate analysis of appetitive, aversive, and neutral uncertainty in GLM2 specified the findings of GLM1. Uncertainty coding occurred similarly for appetitive and aversive outcomes in cuneus and left lOFC. In contrast, activity in right lOFC, MOG, and IOG preferentially encoded aversive uncertainty. ***c***, Generic uncertainty signals (identified with a parametric modulator formed according to Fig. 1*g*). More uncertain neutral outcomes activated particularly occipital regions (Calcarine_L, *t *= 11.07; Lingual_R, *t *= 5.99). All tests of parametric modulations against zero in ***a*** and ***c*** were whole-brain FWE cluster-corrected (*p *< 0.05; cluster-forming threshold, *p *< 0.001). Direct whole-brain comparisons (i.e., not on the extracted data) between parametric modulators showed a statistically (*p *< 0.001, uncorrected) stronger relation of activity to valenced than generic uncertainty in the regions shown in ***a*** and to generic than valenced uncertainty in the regions shown in ***c***. ***d***, Similar coding of uncertainty in all three domains occurred in the calcarine and the lingual gyrus. *p < 0.05; n.s., not significant. All coordinates for data extraction were determined with leave-one-subject-out analysis using GLM1. Error bars represent the standard error of the mean.

The parametric interrogation of the valenced uncertainty model by GLM1 revealed increasing activity (Fig. 5*a*) in the right dorsal anterior insula (*t* = 6.01), bilateral regions of the lateral orbitofrontal cortex (lOFC; left, *t* = 5.96; right, *t* = 5.06), and occipital cortex [cuneus, *t* = 13.03; middle occipital gyrus (MOG), *t* =5.36; IOG, *t* = 4.39; Table 3. All data were cluster-level corrected, *p* < 0.05; cluster-inducing voxel-level threshold: *p* < 0.001]. Direct comparisons within SPM showed that the positive relation of neural activity to valenced uncertainty was significantly stronger than the relation with generic uncertainty in all these regions (*p* < 0.001, uncorrected). Together, these data suggest a specific relation of the occipital and orbitofrontal cortices to the uncertainty of motivationally relevant events rather than uncertainty (risk) as such.

**Table 3.** Brain regions encoding valenced uncertainty and generic uncertainty (GLM1)

Contrast	Region	Cluster size (# voxels)	Mean *t* statistic	Peak MNI coordinates		
				*x*	*y*	*z*
Valenced uncertainty	Cuneus_R (occipital cortex)	3,236	13.03	14	−90	16
	Calcarine_L (occipital cortex)		7.97	0	−86	−4
	Occipital_Mid_L (MOG)		6.78	−18	−92	16
	Right cerebral white matter^a^, extending into the right dorsal anterior insula	153	6.01	24	28	0
	Frontal pole^a^, extending into the lOFC	102	5.96	−22	36	−24
	Frontal pole^a^, extending into the lOFC		4.16	−18	58	−20
	Right cerebral white matter^a^	187	5.39	16	16	26
	Occipital_Inf_R (IOG)	196	5.39	32	−90	−10
	Occipital_Mid_L (MOG)	88	5.36	−34	−88	14
	Frontal pole^a^	66	5.18	40	62	−8
	OFCant_R (lOFC)	108	5.06	18	56	−18
	OFCant_R (lOFC)		5.00	26	38	−22
	Right cerebral white matter^a^	91	4.93	22	44	−2
	Left cerebral white matter^a^	85	4.66	−8	14	22
	Occipital_Inf_R (IOG)	80	4.39	44	−72	−10
	Insula_R (posterior insula)^b^	69	4.11	44	−4	2
Generic uncertainty	Calcarine_L (occipital cortex)	843	11.07	−16	−98	−6
	Lingual_R (occipital cortex)	420	5.99	22	−96	−10

Activations were whole-brain family-wise rate error (FWE) cluster-level corrected, *p* < 0.05; cluster-inducing voxel threshold *p* < 0.001. Apart from a (see below), the gray matter labels are from the Automated Anatomical Labelling (AAL) Atlas 3 (Rolls et al., 2020).

Abbreviations: MNI, Montreal Neurological Institute; MOG, middle occipital gyrus.; lPFC, lateral prefrontal cortex; lOFC, lateral orbitofrontal cortex; IOG, inferior occipital gyrus.

c Label from Harvard-Oxford Atlas (region not defined in the AAL Atlas 3).

d Closer inspection of condition-specific insula activity with GLM2 revealed a combination of valenced inverse probability and neutral precision rather than valenced uncertainty proper (data not shown).

Using GLM2, we again asked whether the relations of putative valenced uncertainty regions were common for the two valenced domains. The valenced uncertainty signals in the right insula, left lOFC, and cuneus identified by GLM1 arose similarly for uncertain appetitive and aversive outcomes (Fig. 5*b*). Moreover, in line with proper valenced uncertainty signals, these regions encoded the uncertainty of the two valenced outcomes more strongly than that of neutral outcomes, although for the left lOFC, the difference was only at trend-level (*p* = 0.06). Separate analyses of valenced uncertainty and nonvalenced uncertainty corroborated this conclusion (data not shown). In contrast, the right lOFC and different occipital regions encoded primarily the uncertainty of aversive outcomes rather than the uncertainty of appetitive outcomes (Fig. 5*b*). Thus, as with probability, some apparent valenced uncertainty signals were in fact driven by a single domain. Moreover, the confirmation of proper valenced uncertainty signals indicates that the motivational nature of outcomes matters for the processing of uncertainty.

We used inclusive masking to assess the overlap of whole-brain corrected valenced uncertainty and probability signals. The only brain regions that commonly encoded valenced probability and uncertainty were in the medial occipital cortex (parts of calcarine, cuneus, and lingual gyrus; Figs. 4*a*, 5*a*). Thus, while valenced probability and uncertainty can be coencoded, separate signals appear to be the norm.

#### The occipital cortex processes generic uncertainty

To investigate neural representations of generic uncertainty not limited to cues predicting appetitive or aversive liquids (Fig. 1*g*), GLM1 interrogated a parametric modulator that increased with uncertainty also for cues predicting neutral liquid. This regressor identified whole-brain FWE cluster-corrected (*p* < 0.05; cluster-inducing voxel-level threshold, *p* < 0.001) generic uncertainty signals only in the bilateral occipital cortex (left calcarine, *t* = 10.41; right lingual gyrus, *t* = 5.80; Fig. 5*c*. Both areas overlapped with occipital regions encoding generic probability). Direct comparisons within SPM showed that the relation with generic uncertainty was significantly stronger than with valenced uncertainty in both regions (*p* < 0.001, uncorrected). Separate analyses of valenced and neutral conditions provided converging evidence in that both areas showed significant uncertainty coding both for cues predicting valenced outcomes and for cues predicting nonvalenced outcomes (data not shown). GLM2 found no significant differences between activations related to the uncertainty of appetitive, aversive, and neutral outcomes (Fig. 5*d*), in keeping with a proper generic uncertainty signal. In conclusion, generic uncertainty coding was more restricted than generic probability coding, and the occipital cortex played a role in processing both generic probability and uncertainty.

#### Occipital cortex activity related to generic probability and uncertainty independent of the pupil diameter

**Figure 6.**
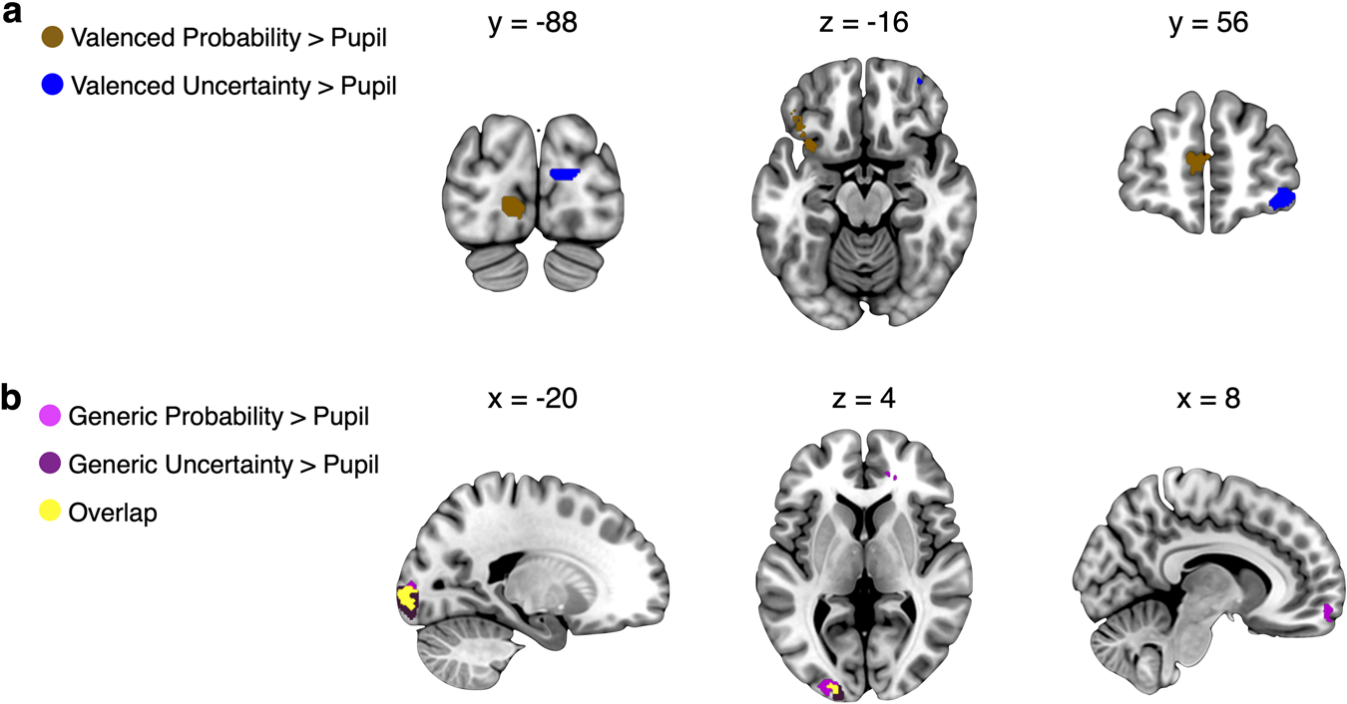
Comparison of probability and uncertainty versus pupil diameter (GLM1). **a**, Neural activity showed significantly stronger relations to valenced probability (brown) than pupil dilation in the left ventral anterior insula (*t *= 5.34), mPFC (*t *= 5.33), left lOFC (*t *= 4.14), and left occipital cortex (*t *= 7.54). Conversely, valenced uncertainty (blue) activated the right lPFC (*t *= 5.88) and right occipital cortex (*t *= 7.38) significantly more strongly than pupil dilation. ***b***, Compared to pupil dilation, generic probability (dark pink) activated the anterior vmPFC (avmPFC; *t *= 5.34), and left occipital cortex (*t *= 6.70) significantly more strongly. Generic uncertainty (purple) was encoded significantly more strongly than pupil dilation in the left occipital cortex (*t *= 9.71). Neural activations are whole-brain FWE cluster-level corrected (*p *< 0.05; cluster-inducing voxel-level threshold, *p *< 0.001).

One may wonder whether the relationship between central occipital activity and both generic probability and uncertainty simply reflected effects on pupil dilation (Figs. 4*c*, 5*c*). This appeared not to be the case. Although pupil dilation correlated with occipital activity, the relation of occipital activity to generic probability and uncertainty was significantly stronger than to pupil dilation (whole-brain cluster-level corrected at *p* < 0.05; cluster-inducing voxel-level threshold at *p* < 0.001; for generic probability in MOG, *t* = 6.70; for generic uncertainty in calcarine, *t* = 9.71; Fig. 6; see Table 4 for a direct comparison in SPM, not based on extracted *β*). Thus, the occipital effects reported above reflected not only changes in pupil dilation, a finding that further extends the multiplexed nature of occipital probability and uncertainty signals (generic more posterior, valenced more anterior).

**Table 4.** Brain regions showing a stronger relation to different forms of probability and uncertainty than pupil dilation (GLM1)

Contrast	Region	Cluster size (# voxels)	Mean *t* statistic	Peak MNI coordinates		
				*x*	*y*	*z*
Valenced probability—pupil dilation	Lingual_L (occipital cortex)	118	7.54	−10	−86	−2
	Insula_L (ventral anterior insula)	140	5.34	−34	14	−16
	OFClat_L (lOFC)		4.14	−48	32	−14
	Frontal_Sup_Medial_L (mPFC)	120	5.33	−8	54	12
Valenced uncertainty—pupil dilation	Cuneus_R (occipital cortex)	82	7.38	14	−90	16
	Frontal pole^a^ (lPFC)	89	5.88	40	62	−8
	Left cerebral white matter^a^	73	5.05	−8	0	24
Generic probability—pupil dilation	Occipital_Mid_L (MOG)	251	6.70	−24	−96	4
	Right cerebral white matter^a^	145	5.56	24	38	0
	Frontal_Med_Orb_R (avmPFC)	69	5.44	8	62	−12
Generic uncertainty—pupil dilation	Calcarine_L (occipital cortex)	406	9.71	−16	−98	−6

Activations are whole-brain family-wise rate error (FWE) cluster-level corrected, *p* < 0.05; cluster-inducing voxel threshold *p* < 0.001.

Abbreviations: MNI, Montreal Neurological Institute; lOFC, lateral orbitofrontal cortex; mPFC, medial prefrontal cortex; lPFC, lateral prefrontal cortex; MOG, middle occipital gyrus; avmPFC, anterior ventromedial prefrontal cortex. Apart from ^a^ (see below), the gray matter labels are from the Automated Anatomical Labelling (AAL) Atlas 3 (Rolls et al., 2020).

c Label from Harvard-Oxford Atlas (region not defined in the AAL Atlas 3).

#### Inverse probability signals in the parahippocampal region

**Figure 7.**
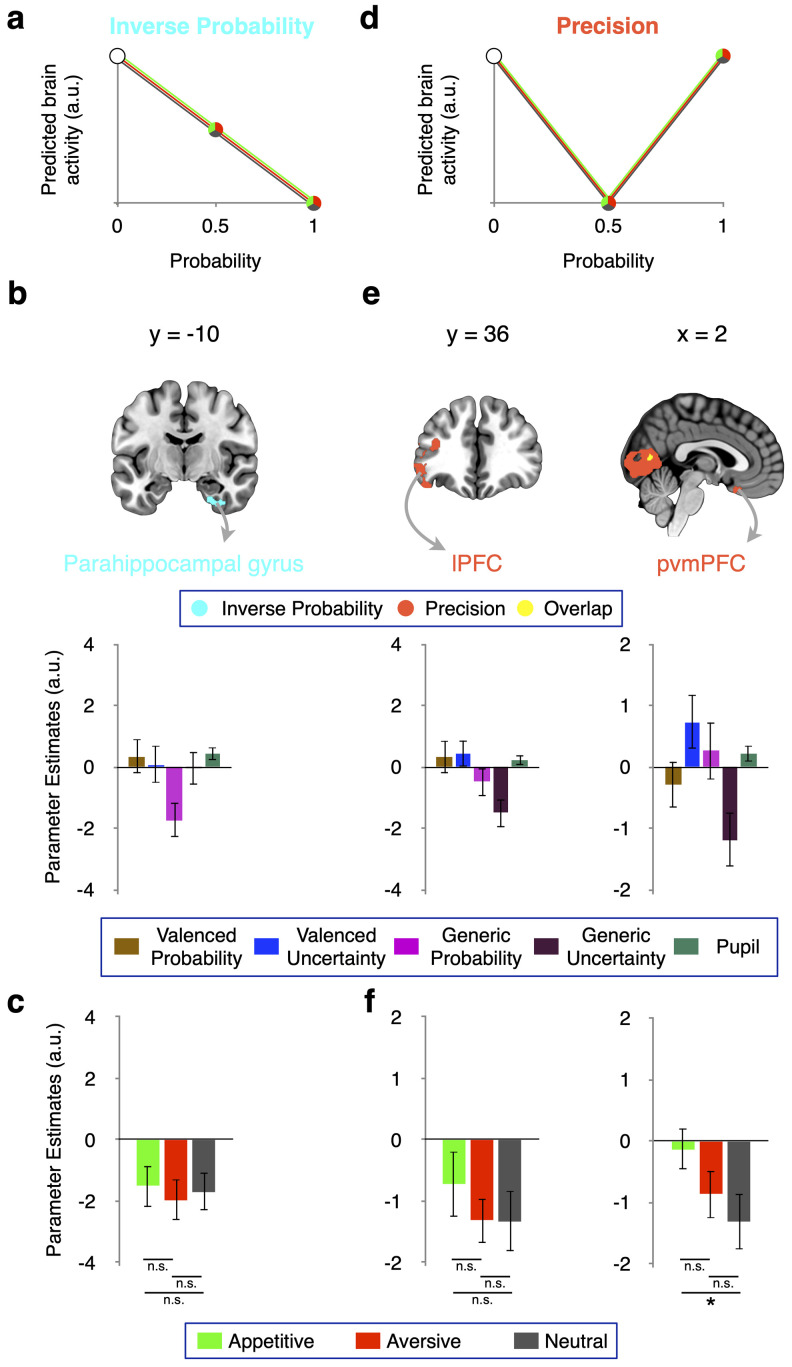
Inverse probability and precision: schematic and signals. ***a***, Predicted brain activity for inverse probability decreased with increasing generic probability. ***b***, Inverse probability signal in parahippocampal gyrus (*t *= 6.20; identified by weighting the generic probability parametric modulator in GLM1 with a −1). In direct whole-brain comparisons activity in this region showed a significantly more negative relation to generic than to valenced probability (*p *< 0.001, uncorrected). ***c***, Separate analysis of appetitive, aversive, and neutral inverse probability in GLM2 validated the findings of GLM1. Proper inverse probability signals require a similar negative relation to probability in all three domains. These requirements were not imposed by GLM1 but met by the parahippocampal gyrus. ***d***, Predicted brain activity for precision was minimal at *p *= 0.5 and maximal at *p *= 0 and *p *= 1, i.e., the inverse of generic uncertainty. ***e*** Precision signals in lPFC (*t *= 6.16) and posterior vmPFC (pvmPFC; *t *= 5.11). Direct whole-brain comparisons (i.e., not on the extracted data) between parametric modulators showed a statistically (*p *< 0.001, uncorrected) stronger relation of activity to generic than valenced precision in these regions. ***f***, Proper generic precision signals require similar inverse relations of brain activity to uncertainty in all three domains. This requirement was met by the lPFC, but not by pvmPFC, which showed preferential coding of neutral precision. All tests of parametric modulations against zero in ***b*** and ***e*** were whole-brain FWE cluster-corrected (*p *< 0.05; cluster-forming threshold, *p *< 0.001). *p < 0.05, n.s., not significant. All coordinates for data extraction were determined with leave-one-subject-out analysis using GLM1. Error bars represent the standard error of the mean.

In models of attention-based learning, motivational salience is computed with absolute errors in the prediction of the outcome (│outcome – prediction│), where prediction increases with probability. In principle, a neural system implementing generic prediction errors could use inverse generic probability signals, which are maximal for cues predicting the absence of valenced and nonvalenced outcomes with certainty and minimal for cues predicting the occurrence of outcomes with certainty (Fig. 7*a*). However, it is unclear whether the brain represents inverse generic probability. We therefore searched for such representations, i.e., an inverse relationship of brain activity with the generic probability parametric modulator of GLM1. We found whole-brain FWE cluster-corrected generic inverse probability signals in the parahippocampal gyrus (*t* = 6.20; Fig. 7*b*, Table 5). Activation in this region decreased similarly with appetitive, aversive, and neutral probability (Fig. 7*c*), in line with full inverse generic probability coding.

**Table 5.** Brain regions with inverse probability and precision (GLM1)

Contrast	Region	Cluster size (# voxels)	Mean *t* statistic	Peak MNI coordinates		
				*x*	*y*	*z*
Inverse probability	Parahippocampal gyrus, anterior^a^	125	6.20	24	−10	−34
	Lingual_R (occipital cortex)	174	5.18	10	−70	2
	Calcarine_L (occipital cortex)	75	4.86	−10	−90	4
Precision	Calcarine_R (occipital cortex)	5,494	14.50	12	−88	12
	Cuneus_L (occipital cortex)		12.63	−8	−90	16
	Lingual_L (occipital cortex)		11.46	−4	−66	4
	Frontal pole^a^	561	6.80	−40	46	−18
	Frontal_Inf_Tri_L (lPFC)		6.14	−50	26	10
	Frontal_Mid_2_L (lPFC)	102	6.16	−36	36	20
	OFCmed_L	60	5.15	−18	20	−22
	Rectus_L (pvmPFC)	75	5.11	0	20	−22
	Temporal_Mid_R	154	5.05	56	−44	−8
	Right cerebral white matter^a^	242	4.89	38	−68	−4
	Occipital_Mid_R (MOG)		4.52	48	−78	14

Activations are whole-brain family-wise rate error (FWE) cluster-level corrected, *p* < 0.05; cluster-inducing voxel threshold *p* < 0.001. Apart from a (see below), the gray matter labels are from the Automated Anatomical Labelling (AAL) Atlas 3 (Rolls et al., 2020).

Abbreviations: MNI, Montreal Neurological Institute; lPFC, lateral prefrontal cortex; pvmPFC, posterior ventromedial prefrontal cortex; MOG, middle occipital gyrus.

a Label from Harvard-Oxford Atlas (region not defined in the AAL Atlas 3).

#### Precision coding in the lateral PFC and posterior vmPFC

Finally, we investigated whether precision, the inverse of generic uncertainty, was represented in the brain. In our task, precision was highest for cues that predicted outcomes with certainty (*p* = 0 and *p* = 1), and it was lowest when uncertainty was greatest (*p* = 0.5; Fig. 7*d*). Accordingly, we looked for activity that exhibited an inverse parametric relationship with the generic uncertainty modulator in GLM1. We identified whole-brain FWE cluster-corrected precision signals (Fig. 7*e*) in the lPFC (*t* = 6.80) and posterior vmPFC (*t* = 5.11). GLM2 revealed similar inverse relations to appetitive, aversive, and neutral uncertainty in the lPFC but little inverse relation to appetitive uncertainty in the pvmPFC (Fig. 7*f*). Thus, the domain-wise analysis specified lateral prefrontal but not posterior ventromedial prefrontal activity as a full generic precision signal.

## Discussion

Our study demonstrates that the brain represents valenced and generic probability and uncertainty. The generic representations occurred in the frontal and occipital cortices and showed increasing activity levels for cues associated with higher probability or uncertainty of any outcome, regardless of valence. Moreover, they were anatomically distinct from valenced probability (mPFC, occipital cortex, and ventral anterior insula) and uncertainty (dorsal anterior insula, OFC, and occipital cortex) signals. At the behavioral level, the effects of probability were predominantly driven by valenced outcomes, suggesting that probability may be particularly sensitive to biological relevance. Conversely, for uncertainty, the generic model tended to explain pupil dilation data better than the valenced model. Thus, uncertainty appears to be associated more easily with nonvalenced outcomes, compatible with an information-processing account.

We note that we operationalized valenced probability by absolute value, as measured by the willingness to pay and rating of each participant. These measures are subjective, and we thereby go beyond previous research that used objective definitions of absolute value and assumed linearity of subjective appetitive and aversive value around zero (Kahnt and Tobler, 2013; Kahnt et al., 2014). Nevertheless, future work may also want to consider alternative measures to equate the psychological or biological salience of appetitive and aversive outcomes. Another limitation of our study is that our FOV covered only the ventral brain. This is particularly relevant for parietal regions, which have been implicated in magnitude-based salience processing (Kahnt and Tobler, 2013; Kahnt et al., 2014), and for the dorsal anterior cingulate, a major hub of the salience network in addition to the insula (Menon, 2015; Uddin, 2015; Seeley, 2019).

### Behavior: preferential effects of valenced rather than generic probability

Cues with higher valence probability induced faster responses and increased pupil size (Fig. 2), suggesting that for probability, primarily valenced outcomes bestow cues with the power to attract attention. These findings converge with reports of larger or more predictable valenced outcomes reducing response times in animals (Roesch and Olson, 2004; Lin and Nicolelis, 2008; Matsumoto and Hikosaka, 2009; Avila and Lin, 2014) and humans (Kahnt and Tobler, 2013; Fontanesi et al., 2019). Thus, whether outcomes are valenced matters for driving behavior, over and above the coding of predictive information (Rao and Ballard, 1999; Feldman and Friston, 2010) about probabilistic generic outcomes. This finding is in line with the notion (Kahnt and Tobler, 2017) that directing attention preferentially to predictors of survival-relevant outcomes is adaptive.

In contrast, a higher probability of neutral outcomes had no (response time) or very weak (pupil diameter) effects on our behavioral measures, suggesting that valenced rather than generic probability drives pupil size. Thus, the consistent occurrence of a neutral outcome (liquid) failed to substantially increase attention to our task. This finding potentially sheds new light on latent inhibition (Lubow and Moore, 1959; Rodríguez et al., 2019; Miller et al., 2022), i.e., the well-documented finding that cues consistently followed by no outcome elicit less attention and subsequently enter associations less easily. Our findings suggest the testable hypothesis that it is not just the absence of an outcome but the absence of an outcome with motivational relevance that produces the latent inhibition effect.

### Behavior: the pupil processes inverse generic uncertainty

Uncertainty paints a different picture. Generic uncertainty decreased rather than increased pupil size and moderately accelerated cue-induced responding. Thus, purely generic uncertainty apparently can affect behavior. Interestingly, pupil dilation correlated with the inverse of generic uncertainty, i.e., precision. Thus, higher uncertainty was associated with smaller pupils. This finding may appear surprising given that pupil dilation has typically been associated with surprise and uncertainty (Joshi and Gold, 2020; Dobbins, 2021). However, it is important to keep in mind that our participants had well learned the meaning of all cues. Accordingly, the uncertainty was expected rather than unexpected (Dayan and Yu, 2006) in our task. By extension, expected and unexpected uncertainty may drive some measures of attention differently.

### Generic probability coding in medial prefrontal and generic uncertainty as well as in the occipital cortex

We found a novel generic probability signal in the mPFC, which was separate from valenced probability and value signals within the mPFC (Figs. 3, 4). Thus, the processing of outcomes in a common currency by the vmPFC extends from value (Bartra et al., 2013) to probability. Probability provides an important organizational principle for cue–outcome relationships, and it is tempting to speculate that some of the decision-making deficits of patients with vmPFC lesions (Noonan et al., 2017; Spaniol et al., 2019) may be due to impaired probability processing.

Both generic probability and uncertainty activated the occipital cortex. Both were cue-induced, eliciting top-down attention, and thereby should facilitate the processing of sensory information. For visual information, this facilitation may be implemented through changes in pupil size. It is therefore noteworthy that the effects of probability and uncertainty (as well as precision) on occipital activity were stronger than, as well as coexistent with, the previously documented (Yellin et al., 2015) effects of pupil size on occipital activity. In other words, the occipital cortex appears to be exquisitely sensitive to top-down generic attention and ideally posed for modulating visual input.

The finding of common occipital cortex coding of generic probability and uncertainty (Fig. 6), over and beyond encoding of pupil activity, extends the theoretical predictions of a model that combines valenced probability and uncertainty (Esber and Haselgrove, 2011). Our findings indicate that as with valenced outcomes (Esber and Haselgrove, 2011; Kahnt and Tobler, 2017), probability and uncertainty processing are also not mutually exclusive for general outcomes. Accordingly, both strong (*p* = 1) and weak (*p* = 0) predictors of any kind of outcomes induce weaker activity in the calcarine and lingual gyrus (Fig. 5*c*) than intermediate (*p* = 0.5) predictors, while strong predictors (*p* = 1, *p* = 0.5 > *p* = 0) induce stronger activity than weak ones. In agreement with an informational account, the activity of these occipital areas combines probability and uncertainty and is independent of outcome valence.

### Neural dissociation of valenced and generic variables

Supporting our hypothesis, ventral and more anterior regions of the mPFC encoded the probability of any outcome, and dorsal and more posterior regions encoded specifically the probability of valenced outcomes (Fig. 4*a*). Our results extend prior demonstrations that the value of higher-order reinforcers (money) is encoded in anterior regions of the PFC, whereas that of primary reinforcers (e.g., sexual stimuli and appetitive liquids) is processed in more posterior regions (Prévost et al., 2010; Sescousse et al., 2010, 2013; Metereau and Dreher, 2015). Moreover, they converge with, and expand upon, reports of valenced probability in monkey mPFC (Monosov and Hikosaka, 2012). Together with the previous research, our findings suggest that the functional specialization within the PFC concerns not only value but also generic versus valenced probability.

Both valenced and generic signals were processed in the occipital cortex (Figs. 4, 5), with posterior regions preferentially encoding generic forms and anterior regions preferentially processing motivational forms. These findings point to the importance of studying not only appetitive and aversive outcomes but also neutral outcomes. With regard to appetitive outcomes specifically, our findings raise the question of whether some of the previously reported relations of occipital cortex activity in the appetitive domain (Serences, 2008) may actually have been due to (valenced or generic) probability. More generally, our findings show that valenced and generic forms of probability and uncertainty can be conceptually and empirically distinguished.

**Figure 8.**
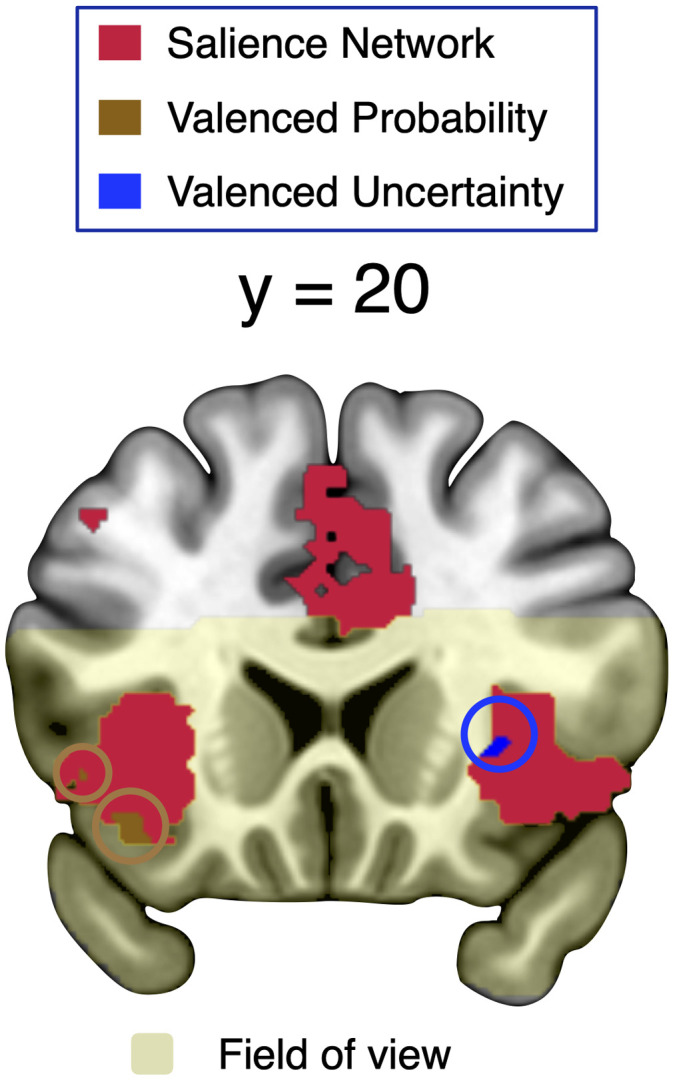
Relation of insula findings to salience network. Valenced probability signals in more ventral anterior insula and valenced uncertainty signals in more dorsal anterior insula located within insula regions identified by the term “salience network” in Neurosynth (red). The field of view of the present study is indicated in light yellow.

The insula encoded valenced probability in ventral anterior regions (Fig. 4*a*) and valenced uncertainty in dorsal anterior regions (Fig. 5*a*; in line with reports on monetary risk signals in the insula: Bossaerts, 2010; Rudorf et al., 2012). Moreover, activity in the posterior insula decreased with valenced probability. Together, our findings add a novel dimension to previous reports of functional parcellation of the insula (Craig et al., 2000; Chang et al., 2013; Geuter et al., 2017; Fazeli and Büchel, 2018). In addition, exploratory analyses showed that all these insula clusters fell within insula regions defined by the term “salience network” (Menon, 2015) in Neurosynth (Fig. 8). This finding highlights the importance of formally defining salience and indicates that the insula codes multiple forms of salience (in the sense of valenced probability and valenced uncertainty). Interestingly, the insula did not show a significant relation to the probability or uncertainty of neutral liquid (in contrast to the vmPFC and occipital cortex), in line with a preferential role of the insula in processing motivationally relevant inputs, such as pain and taste.

## Conclusions

Our study shows that the human brain processes generic probability (anterior vmPFC, posterior ventromedial occipital cortex) and generic uncertainty (lateral OFC, central occipital cortex) signals and that generic variables are represented largely separately from valenced variables. Indeed, even the occipital cortex distinguished between valenced and generic forms of probability and uncertainty. In keeping with the importance of valenced outcomes (ultimately for survival), the neural representation of uncertainty was preferentially valenced rather than generic.
